# Mini Review: Current Trends and Understanding of Exosome Therapeutic Potential in Corneal Diseases

**DOI:** 10.3389/fphar.2021.684712

**Published:** 2021-08-19

**Authors:** Anil Tiwari, Aastha Singh, Sudhir Verma, Sarah Stephenson, Tuhin Bhowmick, Virender Singh Sangwan

**Affiliations:** ^1^ Department of Cornea and Uveitis, Dr. Shroff’s Charity Eye Hospital, New Delhi, India; ^2^ Department of Zoology, Deen Dayal Upadhyaya College (University of Delhi), New Delhi, India; ^3^ Pandorum Technologies Ltd., Bangalore Bioinnovation Centre, Bangalore, India; ^4^ Department of Surgery/Division of Transplant Surgery, The Medical University of South Carolina, Charleston, SC, United States

**Keywords:** exosomes, cornea, bioengineering, wound healing, biomarker, tear

## Abstract

Exosomes are a subset of extracellular vesicles (EVs) that are secreted by most cell types. They are nanosized EVs ranging from 30 to 150 nm. The membrane-enclosed bodies originate by the process of endocytosis and mainly comprise DNA, RNA, protein, and lipids. Exosomes not only act as cell-to-cell communication signaling mediators but also have the potential to act as biomarkers for clinical application and as a promising carrier for drug delivery. Unfortunately, the purification methods for exosomes remain an obstacle. While most of the exosome researches are mainly focused on cancer, there are limited studies highlighting the importance of exosomes in ocular biology, specifically cornea-associated pathologies. Here, we summarize a brief description of exosome biogenesis, roles of exosomes and exosome-based therapies in corneal pathologies, and exosome bioengineering for tissue-specific therapy.

## Introduction

Exosomes are a class of extracellular vesicles (EVs) with diameters of 30–150 nm that are secreted into the extracellular space from almost all cell types. Cells also secrete other EVs, such as apoptotic bodies and ectosomes (Thery et al., 2002; Kalra et al., 2012). Exosomes are commonly found in several biological fluids, such as urine, serum, breast milk, tear fluids, vitreous humor, aqueous humor, and saliva, in both homeostatic and pathological conditions (Ibrahim and Marban, 2016). Exosomes have a double-layered membrane structure that acts as a pit for bioactive molecules like DNA, RNA, proteins, and lipids. The content of exosomes induces a functional response in the recipient cells that are cell- and context-dependent. Exosomes act as potent mediators of cell-to-cell communication, which regulate several cell signaling pathways under both physiological and pathological angiogenesis, immunosuppression, and cancer (Maia et al., 2018). The International Society for Extracellular Vesicles (ISEV) describes Minimal Information for Studies of Extracellular Vesicles (“MISEV”) guidelines for the field in 2014. As per ISEV, the minimal set of information for studying EVs is separation/isolation, characterization, and functional studies (Thery et al., 2018).

The unique structure of exosomes consists of both proteins and lipids. Exosomes are mainly composed of transport proteins, fusion proteins, heat shock protein (HSP), CD9, CD81, and phospholipid-related proteins, all of which can act as markers for exosome selection and characterization (Zhang et al., 2019). Mass spectrophotometric results indicate the presence of nearly 4400 different proteins within the exosome. The large variety of proteins ultimately make exosomes an ideal candidate as the cargo delivery vehicle for intracellular communication. These unique properties of exosomes provide opportunities for innovations in diagnosis, treatments, and drug delivery (Li et al., 2019a). For instance, exosomes may contribute to the propagation of certain diseases including cancer metastasis; like tumor cells, exosomes act as a proangiogenic factor (Ahmadi and Rezaie, 2020); the addition of diabetic sera disrupts the mesenchymal stem cell-derived exosomes (MSC-Exos) signaling (Rezaie et al., 2018), stroke pathogenesis (Mahjoubin-Tehran et al., 2021), gynecological cancer (Hashemipour et al., 2021), cervical cancer (Nahand et al., 2020), malignant glioma (Ghaemmaghami et al., 2020), and sepsis (Hashemian et al., 2020). They can also be used extensively in regenerative medicine; therefore, understanding the content, biogenesis, and release mechanisms of exosomes will enhance our understanding of pathologies and provide insight for new treatment options (Azmi et al., 2013; Basu and Ludlow, 2016). Due to their nanoscale size, exosomes could potentially be used as drug delivery particles for specific targeting with minimal toxicity. Recently, MSC-derived exosomes were also shown to be effective against SARS-CoV2 pneumonia (Akbari and Rezaie, 2020) and osteoarthritis (Mianehsaz et al., 2019).

The ocular system is one of the major sensory systems. Many signaling pathways, such as the Wnt, TGF-β, and FGF pathways are involved in the development of the ocular system (Heavner and Pevny, 2012). Some contributing factors that can lead to vision loss include trauma, aging, and hereditary factors. Recent studies have shown that exosomes are secreted by ocular cells ((Han et al., 2017). Vision impairment, including blindness, is an important public health concern and it is reported that nearly 2.2 billion people are affected by vision impairment or blindness worldwide, affecting primarily the middle-aged and elderly population (Sabanayagam and Cheng, 2017; Bourne et al., 2018). As per 2019, the World Health Organization reported that the leading causes for vision impairment are uncorrected refractive error, cataract, diabetic retinopathy, age-related macular degeneration, glaucoma, and corneal opacity. The cornea refers to the transparent front surface of the eye, which is characterized by its powerful refractive ability and considered a key component of the optical system. Thus, diseases of the cornea are also contributing factors to vision impairment (Flaxman et al., 2017; Fricke et al., 2018).

There are currently limited therapies for corneal repair including surgery, intraocular injections, and eye drops. These treatment plans primarily focus on suppressing the disease development and progression rather than tissue repair. Therefore, there is a need for research into alternative treatments, including research into regenerative cell-based therapies. With the information derived from exosomes studies in other fields, data suggest the potential of exosomes as a therapeutic potential in corneal-related diseases. While there is extensive literature describing the role of exosomes in cancer, their function in the eye is limited. Here, we review current knowledge of exosome function in the visual system in the context of corneal pathologies. We will also discuss the recent development in the exosome field, including bioengineered exosomes and exosomes as potential biomarkers for disease.

## Biogenesis, Release, and Uptake of Exosomes

**FIGURE 1:**
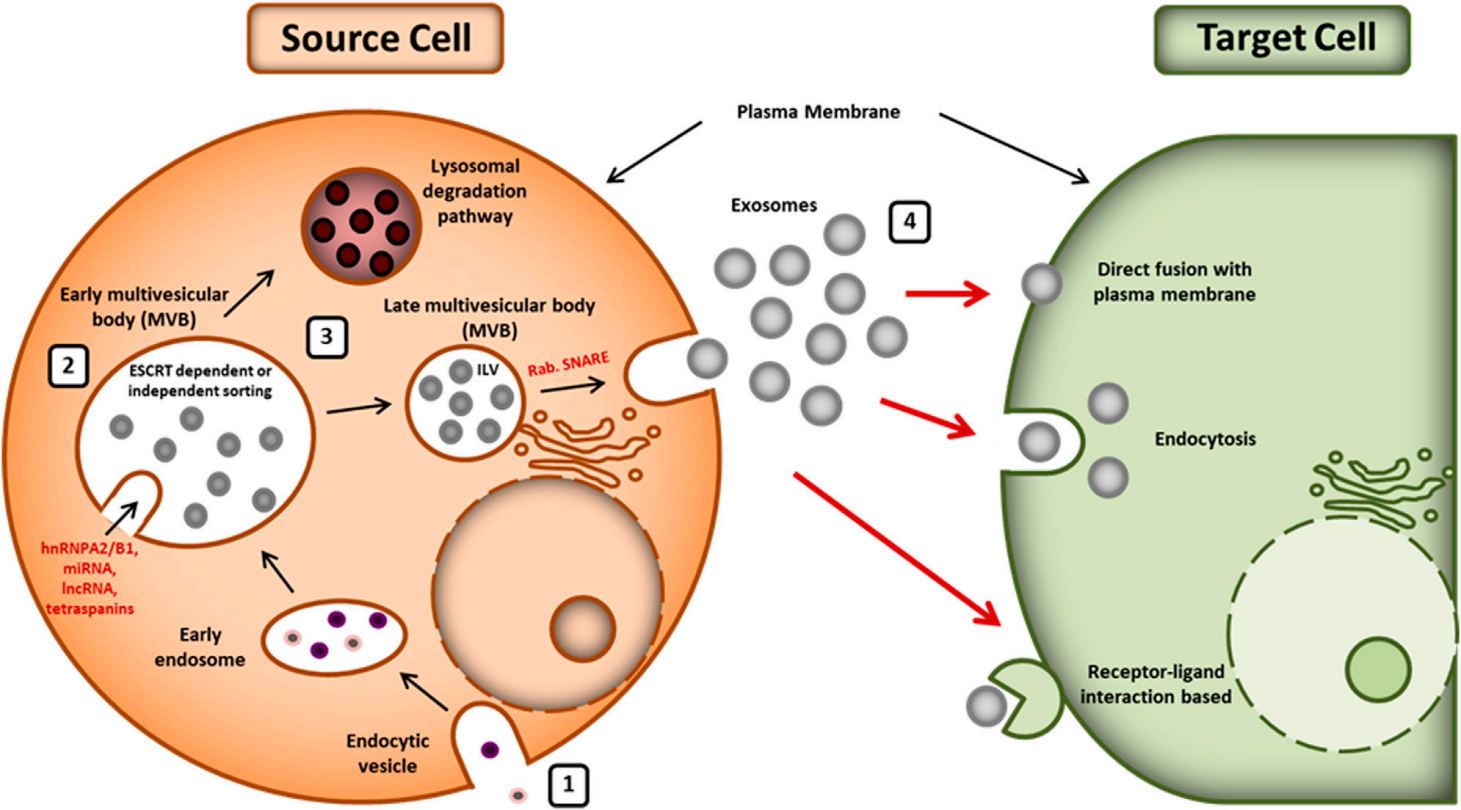
Biogenesis, release, and uptake of exosomes: **(1)** exosome biogenesis starts with the formation of an endosomal vesicle from the plasma membrane. The endocytic vesicle generates ILV, which further forms the MVB. **(2)** Sorting of exosomal content is mediated by various ESCRT-dependent and independent mechanisms. Exosomal cargoes (hnRNPA2/B1, lncRNAs, miRNA, tetraspanins, etc.) approach the MVB membrane in order to be loaded into exosomes. **(3)** The MVBs can either release exosomes outside the cell using Rab and SNARE complex or be degraded by lysosomes. **(4)** The released exosomes can be taken by the recipient cells whether by direct fusion to plasma membrane, by endocytosis, or by ligand-receptor interaction. hnRNPA2/B1: heterogeneous nuclear ribonucleoproteins A2/B1; lncRNA: long noncoding RNA, miRNA: microRNA, ESCRT: endosomal-sorting complex required for transport; MVB: multivesicular body; ILV: intraluminal vesicle; SNARE: soluble N-ethylmaleimide-sensitive fusion attachment protein receptor.

Biogenesis of exosomes is an endosome-dependent, progressive cytological process, which starts with the inward budding of the plasma membrane to form early endosomes. With the maturation of these early endosomes, intraluminal vesicles (ILV) are formed in the lumen of the late endosomes, which are also called multivesicular bodies (MVBs) or multivesicular endosomes (MVEs) (Gurunathan et al., 2019). There are two possible fates of MVBs in the cell: i) fusion with the plasma membrane and release of their internal content in extracellular space as exosomes or ii) fusion with lysosomes with subsequent degradation (Zhang et al., 2019). Figure 1 depicts the biogenesis, release, and uptake of exosomes.

The exosome’s cargo contains different proteins, RNAs, and lipids. Sorting of exosomal content is carried out by various molecular types of machinery such as endosomal sorting complex required for transport (ESCRT) machinery, ESCRT-independent machinery, and other modulatory mechanisms (Villarroya-Beltri et al., 2014; Anand et al., 2019; Wei et al., 2021). ESCRT is an intricate protein machinery that is comprised of four complexes (i.e., ESCRT-0, ESCRT-I, ESCRT-II, and ESCRT-III) with several associated proteins (including VPS4, Tsg101, VTA1, and ALIX) that are highly conserved from yeast to mammals (Colombo et al., 2013; Frankel and Audhya, 2018; Juan and Furthauer, 2018). The ubiquitin binding subunits of ESCRT-0 recognize and sequester ubiquitinated cargo proteins into specific domains of endosomal membranes. ESCRT-I and ESCRT-II form the stable membrane neck and ESCRT-III drives the vesicle neck scission, dissociation, and recycling of the ESCRT-III complex with the energy supplied by Vps4 protein (Henne et al., 2011; Juan and Furthauer, 2018). Alternatively, the exosomes biogenesis and cargo loading are reported to occur in ESCRT-independent way as well (Stuffers et al., 2009). Absence of ESCRT machinery in mammalian cells has been shown to result in impaired cargo sorting into ILVs and variations in ILV number and size but it did not block the formation of MVBs. It suggests that exosomes biogenesis might result from coordinated involvement of both ESCRT-dependent and ESCRT-independent machinery. Various bioactive substances have been reported to regulate the biogenesis and sorting of exosomes such as tetraspanin (Babst, 2011), ceramide (Castro et al., 2014), sphyngosine-1-phosphate (S1P) (Kajimoto et al., 2013), phospholipase D2 (PLD2) (Laulagnier et al., 2004), syndecan-syntenin-ALIX (Baietti et al., 2012), c-Src (Hikita et al., 2019), GTPase Ral (Hyenne et al., 2018), mixed lineage kinase domain-like (MLKL) (Yoon et al., 2017), and small integral membrane protein of the lysosome/late endosome (SIMPLE) (Zhu et al., 2013).

Proteins are sorted in exosomes primarily by ESCRT machinery, lipid-dependent mechanisms, and the tetraspanins (Villarroya-Beltri et al., 2014). RNA loading into exosomes is lipid-mediated. It depends on the affinity of the cellular RNA with the raft-like region of the outer layer of the MVB membrane (Janas et al., 2015). Sumoylated hnRNPA2B1 has been reported to be a key player in sorting of miRNAs into exosomes (Villarroya-Beltri et al., 2013). Additionally, KRAS (Kirsten rat sarcoma viral oncogene homolog) has been shown to play an important role in sorting miRNA in exosomes (Cha et al., 2015). Record et al. have reviewed the type of lipids vectorized by exosomes and described their role in the fate and bioactivity of exosomes (Record et al., 2014).

The release of exosomes requires an array of crucial factors including cytoskeleton, Rab GTPase, and fusion apparatus SNARE (soluble N-ethylmaleimide-sensitive factor attachment protein receptors) complex (Hessvik and Llorente, 2018). Further, the uptake of exosomes by recipient cells can be through endocytosis (clathrin-, caveolin-, or lipid raft-mediated for different cell types), direct fusion with plasma membrane, or receptor-ligand interaction (Liu et al., 2020).

## Exosomes in Ocular Surface Diseases

### Immune-Mediated Diseases

The role of exosomes, released from both nonimmune and immune cells, during physiological and pathological immune responses is widely documented (Robbins and Morelli, 2014). Endogenous secretion as well as exogenous administration of exosomes can elicit and regulate the immune response in a context- and dose-dependent manner. The functional ability of exosomes is ascribed to transfer and presentation of antigenic peptides, regulation of gene expression by exosomal miRNA, and induction of various immune signaling pathways by the exosome surface ligands (Kalluri and LeBleu, 2020). In the present section, we discuss the diverse roles of exosomes in some of the immune-mediated eye diseases.

#### Sjogren’s Syndrome

Sjögren’s syndrome is a chronic autoimmune disorder characterized by lymphocytic infiltration in the lacrimal and salivary glands, leading to eye and oral dryness. The exocrinopathy can result from primary Sjögren’s syndrome (pSS) or it can be secondary to other autoimmune disorders such as systemic lupus erythromatosus (SLE) or rheumatoid arthritis (Huang et al., 2020). The pathogenesis of Sjögren’s syndrome involves activation of both innate and adaptive immune pathways, e.g., B cell-activating factor (BAFF)/BAFF receptor axis, interferon (IFN) signatures, and nuclear factor kappa B (NF-kB) signaling (Sandhya et al., 2017). Prognostic markers for Sjögren’s syndrome include anti-Ro/SSA, anti-La/SSB, and antinuclear autoantibodies (ANA) (Huang et al., 2020). These autoantigens are released by salivary gland epithelial cells *via* exosomes. Hence, exosomes are involved in the presentation of intracellular autoantigens to the immune system driving disease progression in Sjögren’s syndrome. Another study (Gallo et al., 2016) has shown that Epstein Barr Virus- (EBV-) specific microRNA, i.e., EBV-miR-BART13-3p, is significantly elevated in salivary glands (SGs) of pSS patients. The EBV typically infects B cells but not salivary epithelial cells. However, exosomes can transfer EBV-miR-BART13-3p from B cells to salivary epithelial cells. The subconjunctivally administered MSC-Exos have been shown to efficiently alleviate the induced autoimmune dacryoadenitis in rabbit models, which closely mimic human Sjögren’s syndrome. Thus, MSC-Exos show therapeutic effects for Sjögren’s syndrome-induced dry eyes. MSC-Exos execute their modulatory effects on the polarization of lacrimal macrophage and through the enhancement of Th2 and Treg responses *via* targeting of NF-kB signaling (Li et al., 2019a; Huang et al., 2020).

#### Corneal Allograft Rejection and Regeneration

Though the cornea is an immunologically privileged avascular transparent tissue, corneal grafts are rejected due to an allogeneic immune response. The adaptive immune response is initiated following recognition of donor MHC antigens by recipient T cells (Marino et al., 2016). Gonzalez-Nolasco et al. have recently reviewed the role of exosomes in recognition, rejection, and tolerance of allografts (Gonzalez-Nolasco et al., 2018). Another study shows that short collagen-like peptides conjugated to polyethylene glycol (CLP-PEG) exert proregenerating effects through stimulation of EVs production by host cells (Jangamreddy et al., 2018). Additionally, epithelial-derived exosomes are reported to have a potential role in corneal wound healing and neovascularization. Han et al. have shown that epithelial-derived exosomes mediate communication between corneal epithelial cells, corneal keratocytes, and vascular endothelial cells (Han et al., 2017). Also, the limbal stromal cell- (LSC-) derived exosomes contribute to the proliferation and wound healing of limbal epithelial cells (LEC) (Leszczynska et al., 2018). Human corneal mesenchymal stromal cells (cMSC) exosomes are reported to accelerate corneal epithelial wound healing too.

#### Autoimmune Uveitis

Exosomes serve as a crucial player for immune-regulatory functions of retinal pigment epithelial (RPE) cells in uveitis. Exosomes from both nonstimulated and cytokine-stimulated RPE cells inhibit T-cell stimulation and regulate the viability and phenotype of monocytes (Knickelbein et al., 2016). Though specific mechanisms have yet to be explored, the MSC-Exos warrant therapeutic potential for autoimmune uveitis.

### Wound Healing and Neovascularization

The cornea covers the anterior 1/6th of the total surface of the globe and is lined by a nonkeratinized stratified squamous epithelium, which is richly innervated. Other cellular components of the cornea are the stromal keratocytes and endothelial cells. Injury to the cornea initiates a cascade of reactions initiating repair pathways. Depending on the depth of corneal injury, namely, epithelial, stromal, or endothelial, the injured cells propel a healing response. Tissue repair comprises cell migration, transformation, and the release of growth factors, cytokines, integrins, and proteases. Scar formation during the healing process compromises the transparency of the cornea and can lead to a sight-threatening scenario.

After epithelial injury, damaged cells enter a latent phase leading to cellular apoptosis (Liu and Kao, 2015), followed by the migration of adjacent cells towards the site of injury to repair the epithelial layer. The cell migration is initiated by EGF and further facilitated by HGF and KGF, the expression of which is upregulated by PDGF (Klenkler et al., 2007). This phase is followed by the proliferation and differentiation of epithelial cells to restore the cell density, which is driven by the limbal epithelial stem cells. The proliferating cells ultimately undergo attachment to the basement membrane through hemidesmosomes to complete the process of epithelial layer healing.

Similar to the epithelium, the first response after stromal injury is apoptosis of keratocytes triggered by cytokines, such as TNF-a, IL-1, and Fas ligand. The keratocytes first differentiate into fibroblast under the effect of actin and then migrate to the site of injury where they further differentiate into myofibroblasts (Miyagi et al., 2018). This transformation is initiated by TGF-B1, TGF B2, and PDGF (Miyagi et al., 2018). A makeshift extracellular matrix (ECM) is deposited and the wound closure is attempted. Myofibroblasts express alpha-smooth muscle actin, vimentin, and desmin, which lead to a haze formation. The disorganized ECM and decreased crystallin production can compromise corneal transparency (Ljubimov and Saghizadeh, 2015). Once the barrier function recovers, the level of the aforementioned proinflammatory mediators subsides. HGF helps in the degradation of the ECM, which eventually gets reabsorbed and corneal transparency is reestablished.

Mesenchymal stromal cells have a therapeutic potential to modulate the inflammatory process and promote wound healing. Exosomes have been shown to contribute to this repair process (Li and Zhao, 2014). They effectively inhibit neovascularization, promote clearance of neutrophils, and therefore promote scarless healing of the cornea. After corneal injury, MSCs home to the site of injury under the effect of chemoattractants. MSC migration is mediated by chemokines SDF-1 and substance P released at the site of injury (Yao et al., 2012). In injured corneas, exosomes upregulate the expression of antiangiogenic factors, such as thrombospondin-1 (TSP-1), and anti-inflammatory cytokines, including IL-10, TGF-B1, and IL-6, while downregulating the expression of the proinflammatory factors IL-2, interferon-γ (IFN-γ), macrophage inflammatory protein-1α, and vascular endothelial growth factor (VEGF) (Yao et al., 2012). Ma et al. transplanted human MSCs grown, expanded on the AM into chemically burned corneas of rats, and reported significant improvement in the corneal surface and vision after 4 weeks (Ma et al., 2006). Moreover, studies show a significant reduction in CD45 and interleukin 2 (IL-2) expression in eyes treated with MSCs (Samaeekia et al., 2018). In addition, matrix metalloproteinase-2 (MMP-2), which is associated with inflammation-related angiogenesis, was undetectable in the eyes treated with the MSCs (Samaeekia et al., 2018).

In an *in vitro* study, a monolayer of confluent human corneal epithelial cells (HCECs) scratched and then treated for 24 h with 1.0 × 10^8 exosome/mL media showed a significant increase in the rate of reepithelialization of the monolayer compared to the PBS-treated control (30.1% remaining wound area after 16 h, compared to that of control with 72.9%). Additionally, *in vivo* analysis showed that within 24 h the exosome-treated animals demonstrated significantly greater wound healing compared to control (77.5% healed versus 41.6%) (Samaeekia et al., 2018).

The additional advantages of using exosomes as a therapeutic potential include easy isolation through ultracentrifugation techniques, they do not obstruct small vessels, and they have a low risk of rejection and malignancy. Exosomes can be safely and stably stored as well as their myriad prohealing effects, which can help in scarless wound repair.

### Exosomes as Biomarkers for Eye Diseases

With the advancement of stem cell research in regenerative medicine, the focus has tilted towards utilizing exosomes to identify biomarkers for ocular diseases (van der Merwe and Steketee, 2017). There is an increased potential for the development of exosome-based diagnostic assays. Exosomes and EVs have several features that make them a unique target for finding new biomarkers: i) presence of the lipid bilayer, thereby providing stability and protection of enclosed RNA, DNA, and proteins from nucleases and proteases in the extracellular milieu, ii) exosomes containing tissue-, cell-, or disease-specific proteins and nucleic acids, and iii) the relative tendency to withstand difficult conditions making it possible to use a wide range of methods for isolation and enrichment from a range of biological fluids (i.e., plasma, serum, urine, saliva, semen, breast milk, aqueous humor, and cerebrospinal fluid). Studies from cancer, cardiovascular disease, and diabetes researches report promising findings for the utility of exosome as biomarkers for diagnosis, risk assessment, and a therapeutic vehicle (Lawson et al., 2016; Klingeborn et al., 2017).

Exosomes are produced by healthy and pathological cells. Exosome concentrations have been reported in the serum of cancer patients and have been used for diagnosis and prognosis purposes. Tear fluids also hold potential as a noninvasive source for the identification and characterization of exosome-based biomarkers (Gonzalez and Falcon-Perez, 2015). The biggest challenge associated with analyzing tear exosomes is the sample volume. However, the expression of exosomes markers (CD9; CD63) in tears is significantly higher when compared to serum-derived exosomes, making tears an attractive approach for developing diagnostics (Inubushi et al., 2020). Currently, there is limited literature on the use of tear exosomes as a disease state biomarker; however, tear-based proteomics identified several proteins that are exosome-associated, indicating a need for further exploration (Qin and Xu, 2014; Grigor’eva et al., 2016; Liu et al., 2019). With the advancement in modern technologies and high throughput sequencing facility, the therapeutic potential of exosomes derived from tear samples could be considerable potential as a source for biomarkers.

Exosomes are abundantly present in the ocular fluids such as, tear fluids, aqueous humor (AH), and vitreous humor, all of which are important for maintaining ocular surface health but can also contribute to disease progression (Dismuke et al., 2015; Grigor’eva et al., 2016). Protein profiling of AH and RPE-derived exosomes could be used as a valuable tool for novel diagnostic biomarkers for patients with glaucoma, neovascularized age-related macular degeneration (AMD), and retinal diseases (Kang et al., 2014; Dismuke et al., 2015). Even though there is limited literature, the identification and characterization of exosome-specific biomarkers in eye diseases has great potential. Recently it was shown that vitreous humor exosomes-derived miR-146a could be used as a potential biomarker for uveal melanoma (Ragusa et al., 2015). Though exosomes have been proposed as a potential treatment for ocular surface regeneration and homeostasis in pathologies including corneal fibrosis and dry eye diseases, there are currently, to our knowledge, no reports highlighting the potential use of exosomes derived from pathological tears for diagnostic uses. Moreover, with the recent technological advances in exosome analysis, certainly, this is a wide-open area of research.

## Exosomes Modulation and Corneal Perspective

Exosomes are natural nanomaterials consisting of surface ligands and receptors. Exosomes can be conveniently isolated from cell culture conditioned media and from various biological fluids (Qin and Xu, 2014; Luan et al., 2017; Hessvik and Llorente, 2018). They are superior to synthetic nanovesicles, such as liposomes, owing to their stability, bioavailability, natural origin, circulation half-life, immunomodulatory roles, tissue-specific targeting, and ability to penetrate nonaccessible tissue regions (Antimisiaris et al., 2018). These properties make exosomes an excellent candidate for drug loading, delivery, and site-specific tissue delivery. Due to the lack of tissue and cell-specific targeting features, naturally occurring exosomes face many challenges when being considered as a therapeutic delivery system (Li et al., 2019b), thereby emphasizing the need for modulating the exosomes as per the demand and requirement of pathologies.

Exosome’s engineering has recently been done to modulate exosome behavior to meet the cell, tissue, and pathologic specific demands. Exosomes are engineered at the cellular level under natural conditions but successful exosome modification requires further exploration. Exosomes are secreted from cells expressing lipids, cell surface molecules, and ligands that naturally target specific types of recipient cells. There is evidence pointing to the natural targeting ability of exosomes based on the donor cells. For example, exosomes isolated from neuroblastoma cells express glycosphingolipid glycan groups that have a selective affinity to the amyloid-β aggregates in the brain, providing a promising targeted treatment for Alzheimer’s disease (Hood, 2016). Targeting ligands on the surfaces of exosomes can also be engineered; a widely used strategy is to insert the gene for the targeting ligand into the donor cells. The donor cells will then secrete exosomes expressing the targeting ligand on their surface promoting tissue-specific treatment. A similar approach was used to express Lamp2b in the exosomes of dendritic cells, which was shown to bind strongly to the neuron-specific rabies viral glycoprotein (RVG) peptide. In another study, exosomes were used to deliver let-7a miRNA targeting EGFR-overexpressing breast cancer cells in mice (Alvarez-Erviti et al., 2011; Kesharwani et al., 2012).

Corneal tissue is frequently challenged by biological, chemical, thermal, and traumatic wounds associated with immune cells infiltration, inflammation, neovascularization, and fibrosis. If not treated in a timely manner, the tissue injury could result in loss of vision. Along with MSCs therapy, their secretory products have recently gained interest in corneal regeneration due to their immunomodulatory and antiangiogenic potential (Phinney and Prockop, 2007). The treatment of rabbit corneal stromal cells with adipose MSCs-derived exosomes resulted in increased proliferation, reduced apoptosis, and deposition of ECM (Shang et al., 2020). In another study, topical application of corneal stromal stem cells-derived exosomes suppressed corneal inflammation and fibrosis through a reduction in neutrophil infiltration and fibrotic mediators, tenascin-C, Col3A1, SPARC, etc. in a murine superficial stromal wound model (Lin et al., 2013; Shen et al., 2018). Furthermore, in a mouse model of the corneal wound, results show an increase in wound healing with human mesenchymal stromal cell treatment. All these data taken together indicate a potential role of MSC-derived exosome as a therapeutic potential for corneal disease and further exploration into exosome-based regenerative mechanisms remains.

The function of exosomes is dictated by their cargos, which include small RNAs, specific proteins, lipids, and metabolites. Corneal stromal stem cell- (CSSC-) derived exosomes are proposed to have a unique set of miRNAs that promote corneal integrity, as compared to the exosomes derived from HEK293 cells, justifying the lack of scar-forming activity in the latter set of exosomes. Furthermore, the Funderberg group recently showed that ALIX protein inhibited the packaging of miRNA into exosomes, indicating the regulatory role of ALIX in packaging of miRNA in exosomes. Further, CSSC that expresses lower levels of ALIX is ineffective in reducing scar formation, potentially due to their low cargo packaging efficiency (Hertsenberg et al., 2017). Recently an interesting study from the May Griffith group also reported that exosomes are an important mediator of corneal regeneration. They hypothesized that the presence of exosomes is associated with the production of a new ECM at the surgical site in the rabbits undergoing LiQD cornea treatment compared to the controls (McTiernan et al., 2020).

Currently, studies show that exosomes are modulated to enhance their efficacy to meet the demands of the cell and tissue context. Though there are substantial studies on cancer biology, neurosciences, and immune biology, the concept of exosome remodeling to meet the different aspects of corneal pathologies is still in infancy.

## Future Directions and Concluding Remarks

Exosomes are promising and fascinating biological nanomaterials. With the advancing popularity and promising results, it could be extrapolated that the existing cell therapies could be conveniently replaced by exosome therapy. MSC-Exos paved the path for extensive studies on exosomes, particularly focusing on regenerative medicine. Exosomes have proven to be a promising alternative for cell therapy, as MSCs-derived exosomes have shown to be equally as good as stem cells (Cheng et al., 2017).

**FIGURE 2:**
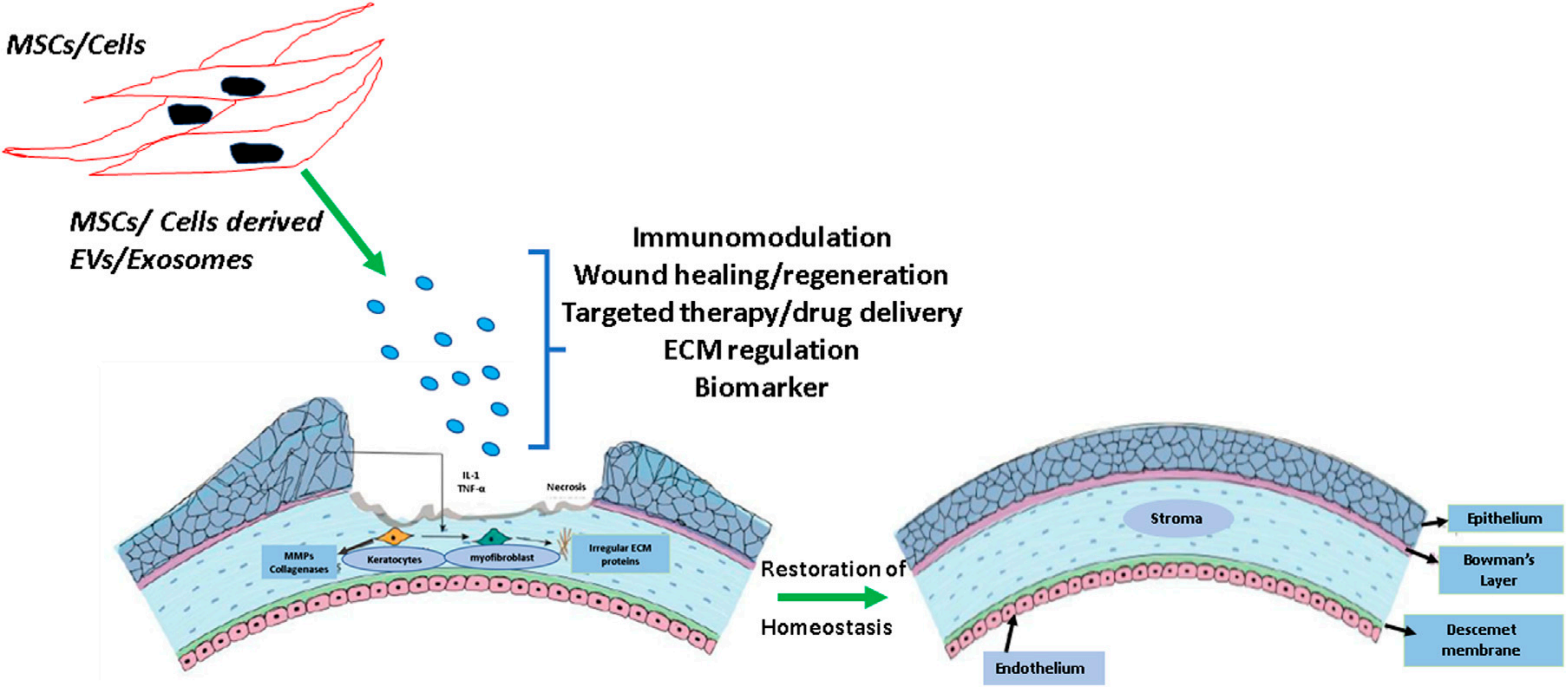
Schematic showing the effect of mesenchymal stem cells (MSCs) and other cell type-derived exosomes on corneal wound healing and fibrosis. Exosomes are actively involved in immunomodulation and extracellular matrix (ECM) remodeling. The use of exosome therapy could be a promising approach to maintain corneal homeostasis and maintaining transparency.

Inflammation, activation of immune cells, neovascularization, and fibrosis are the common disease driving mechanisms of several eye conditions and it is important to note that exosomes have proven to be a promising tool to combat these processes. Exosomes are a promising tool that can take part in immunomodulation, ECM remodeling, and drug delivery (Figure 2). Though there is extensive research highlighting exosome therapy in other fields, sadly, the eye field is trailing in this context. The information given by these fields could be used as a tool for accelerating exosomes and eye research. Using exosomes as biomarkers or therapeutic vehicles holds the potential to lead to better, personalized treatments for patients with eye diseases. This review highlights the immense research opportunities that exist to understand the physiological role and clinical potential of exosomes in ocular health and disease.
